# An Approach for Quantitatively Balancing Methylmercury Risk and Omega-3 Benefit in Fish Consumption Advisories

**DOI:** 10.1289/ehp.1002824

**Published:** 2011-05-04

**Authors:** Alan H. Stern, Leo R. Korn

**Affiliations:** Office of Science, New Jersey Department of Environmental Protection, Trenton, New Jersey, USA

**Keywords:** fish, fish consumption advisories, MeHg, methylmercury, n-3 fatty acids, omega-3 fatty acids, polyunsaturated fatty acids, PUFA, risk–benefit

## Abstract

Background: Nearly all fish consumption advisories for methylmercury (MeHg) are based only on risk. There is a need to also address benefits, especially those from polyunsaturated fatty acids (PUFAs), in neurodevelopmental function and cardiovascular health. However, because MeHg and PUFA generally act on these same end points, disentangling risk and benefit is challenging.

Objectives: We propose an approach for balancing risk and benefit that is based on the use of statistically dissociated measures of risk and benefit.

Discussion: Because of mutual coexposure of MeHg and PUFAs in population-based studies and their opposite effect on many of the same end points, MeHg risk and PUFA benefit are tightly linked statistically, which results in mutual (negative) confounding. Thus, neither MeHg risk nor PUFA benefit can be accurately quantified without taking the other into account. A statistical approach that generates unconfounded risk and benefit coefficients for each end point can permit their subsequent recombination to describe the overall risk–benefit profile of each species of fish or fish diet. However, it appears that some end points may be adversely affected by MeHg without experiencing counterbalancing benefit from PUFAs. Such end points may drive consumption advisories and may preclude balancing of risk and benefit on the basis of other end points.

Conclusions: Our thinking about fish consumption advisories now recognizes the need to balance risk and benefit. However, although statistical analysis of the appropriate data can eliminate mutual confounding, care is required to address the most sensitive end points that may be sensitive to risk and not benefit.

After observations of frank toxicity in the mass poisoning episodes in Japan in the 1950s and in Iraq in the 1970s (World Health Organization 1990), there was a slow realization that lower levels of exposure to methylmercury (MeHg) were also capable of causing more subtle forms of toxicity not readily apparent on an individual, clinical level. Population-based studies have detected neurodevelopmental deficits [National Research Council (NRC) 2000] and probable cardiovascular effects, particularly an increased risk of myocardial infarction (Stern 2005a). Fish consumption advisories for MeHg have been developed based on this understanding and have generally been derived from the reference dose (RfD) for MeHg that addresses neurodevelopmental risk [U.S. Environmental Protection (EPA) 2001]. After realizing that MeHg risk assessment should be broadened to encompass these more subtle forms of toxicity, researchers also recognized that beneficial aspects of fish consumption should be considered when assessing MeHg risk. Although fish provide several beneficial constituents, much of the research on the benefits of fish consumption has focused on polyunsaturated fatty acids (PUFAs) (also referred to as omega-3 fatty acids and n-3 fatty acids), particularly docosahexaenoic acid (DHA) and eicosapentaenoic acid (EPA), which are typical and abundant PUFAs. PUFAs appear to be associated with enhancement of neurodevelopmental function when exposure occurs during gestation and possibly lactation (Cansev et al. 2009; Levant et al. 2010; Mozaffarian and Rimm 2006; Ryan et al. 2010) and with cardiovascular health in adulthood (Mozaffarian et al. 2010; Mozaffarian and Rimm 2006; Roth and Harris 2010; Siddiqui et al. 2009). MeHg produces adverse effects on these same general end points—neurodevelopment (NRC 2000) and cardiovascular health (Stern 2005a; Virtanen et al. 2007). Thus, balancing the risk of MeHg and the benefit of PUFA from fish consumption can be seen, at least in part, as the weighing of competing influences.

In proposing an approach for balancing MeHg and PUFA benefit in the crafting of fish consumption advisories, we focus here on neurodevelopmental risks and benefits because that is the end point addressed by the current U.S. EPA RfD (2001). However, the same considerations would apply in balancing cardiovascular risks and benefits. It is also important to note that for the purposes of this discussion, we are focusing only on MeHg risk and omega-3 benefit. There are other potential contaminants in fish for which the balancing of risks and benefits is not necessarily addressed by the same considerations that apply to MeHg and PUFAs. Likewise, there are potentially other beneficial constituents of fish for which data on benefits are not necessarily confounded by co-occurring contaminants. Because much of the practical discussion about balancing MeHg risk against the benefits of fish consumption has focused on PUFA benefits (Cohen et al. 2005; Guallar et al. 2002; Mozaffarian and Rimm 2006), as have proposals for constructing fish consumption advisories (Ginsberg and Toal 2009; Gochfeld and Burger 2005), we have likewise chosen to focus on these two parameters. However, the following discussion should not be taken to imply that these are the only sources of risk and benefit in fish.

## The Problem

**Figure 1:**
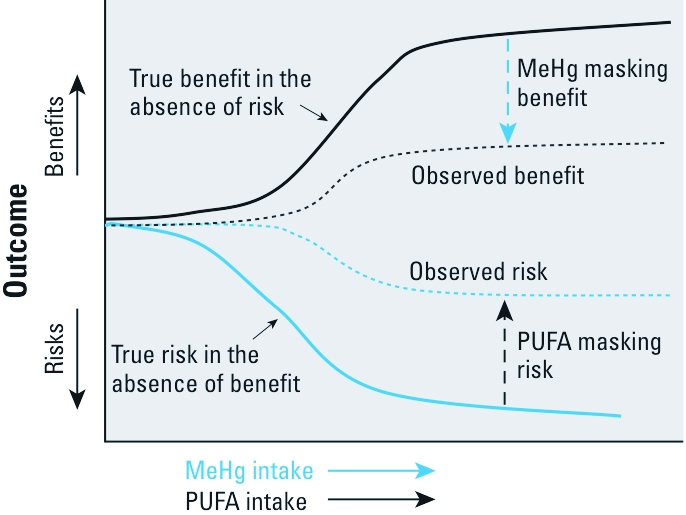
Negative confounding of MeHg risks and PUFA benefits.

**Figure 2:**
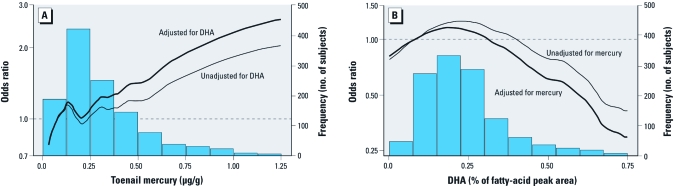
Odds ratios for nonfatal myocardial infarction as a function of mercury exposure (*A*) and DHA intake (*B*), each nonadjusted and adjusted for the negative confounding of the other. Data from Guallar et al. (2002).

Given that MeHg and PUFA operate largely in different directions on the same end points, the analysis of data on risk and/or on the benefit of fish consumption to derive practical fish consumption advisories is not straightforward. This is because in studies of fish-consuming populations, data on the adverse effects of MeHg will be confounded by the simultaneously competing PUFA benefits, and data on PUFA benefits will be confounded by competing MeHg risks. In contrast to classic statistical confounding in which two or more variables affect the outcome in the same direction, this type of confounding with two variables affecting the outcome in opposite directions has been referred to as negative confounding (Choi et al. 2008). Because the major source (and for most populations, the only source) of MeHg exposure is fish consumption and because all fish contain at least some PUFAs, this confounding means that studies of fish consumers will underestimate the underlying risk of MeHg and simultaneously underestimate the underlying benefit of PUFAs (generically represented in Figure 1). For convenience, true and observed benefit and true and observed risk are shown on the same graph in Figure 1, but this should not be taken to imply that observed risk and observed benefit occur simultaneously for the same outcome. Figure 2 presents an empirical example of this from Guallar et al. (2002). Although Figure 2 is derived from myocardial risk–benefit data, the principle it illustrates applies equally to neurodevelopmental risk–benefit analysis.

## How Can We Overcome This Problem to Provide Appropriate Fish Consumption Advice?

Clearly, some combinations of MeHg and omega-3s in fish lend themselves to obvious consumption advice. Fish with characteristically high levels of PUFAs and low MeHg, such as anchovies, sardines, herring, and salmon, will pose net benefits. Conversely, fish with characteristically high levels of MeHg and low levels of PUFAs, for example, swordfish and shark, will pose a net risk. The difficulty arises when we think about how to craft advisories for fish with intermediate levels of MeHg and PUFAs, including tuna, snapper, bluefish, sea bass, freshwater bass, pike, and walleye.

Several studies have regressed data on health outcomes against fish consumption as a whole rather than against PUFAs and MeHg (Daniels et al. 2004; Lederman et al. 2008; Oken et al. 2008a, 2008b). There are two reasons why such an approach should not be used to construct risk–benefit fish consumption advisories. The first is that in almost any fish-consuming population, there will be a variety of patterns of fish consumption. Each pattern will contribute different combinations of MeHg and PUFA intake, but regression of outcome against fish consumption inherently assumes that all consumers eat the same average diet. The second reason is even if it is assumed that a single fish diet is representative of a given population, the risk–benefit balance derived from that population can be applied to a different population only if it is assumed that the second population eats the same fish diet with the same MeHg–PUFA balance; this is by no means an obvious assumption.

Another possibility is to obtain risk information from one study that quantifies MeHg exposure but not PUFA intake and benefit information from a different study that provides data on PUFA intake but not MeHg intake data. Under such a scheme, regression coefficients, BMDLs (benchmark dose–low), or plateau values in a dose–response curve for adverse effects and benefits could be compared. The problem with this approach is that, as represented in Figure 1, PUFA intake–benefit data derived from studies of fish-consuming populations will be at least partly obscured by MeHg effects on the same end point. Similarly, MeHg intake–risk data will be obscured by PUFA effects. Even if the PUFA benefit data are derived from studies in which PUFAs are supplied by dietary supplementation and not from fish, it is difficult to imagine a useful source of MeHg intake–risk data that is not derived from fish consumption with attendant PUFA intake. Thus, even if PUFA-benefit data can be obtained free of MeHg confounding, unconfounded MeHg risk data are not obtainable.

Instead, risk data and benefit data are needed that are derived from the same study in the same population and not obscured by each other. We can think of these unconfounded estimates as naked risk and benefit data.

## Obtaining and Using Unconfounded Risk and Benefit Information

**Figure 3:**
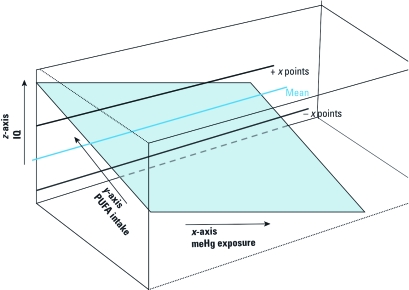
Three-dimensional projection of a hypothetical relationship among IQ, MeHg intake, and PUFA intake with IQ regressed on MeHg and PUFA. The lines show constant values of IQ scores relative to varying levels of MeHg and PUFA. x designates a specific, but indeterminate value.

The data necessary to generate unconfounded risk and benefit information can be derived from well-designed population-based epidemiological studies that generate MeHg and PUFA intake estimates by subject. Intake can be derived from accurate dietary survey data or from MeHg and PUFA biomarker measurements from which intake can be estimated by applying pharmacokinetic models. Models are available that relate MeHg biomarkers (hair and blood) to intake (Shipp et al 2000; Stern 2005b). Relevant pharmacokinetic data are available for PUFAs (Masson et al. 2007; Rusca et al. 2009), but it does not appear that models specifically relating PUFA intake to biomarker concentration have been published to date. Population-based multiple regression (or structural equation) models of appropriate outcomes [e.g., intelligence quotient (IQ)] can then be constructed and are regressed against the independent variables of PUFA and MeHg intake. In multiple regression, the coefficient (β) of each independent variable reflects the slope of the response to each increment of that variable when the slopes of all other independent variables are held constant—when controlling for or adjusting for the other independent variables. Therefore, if we have a regression model for a gestational neurodevelopmental outcome such as IQ with both maternal MeHg intake and maternal PUFA intake as independent variables, the β for each reflects the unconfounded naked effect of each on IQ. In general, for outcomes sensitive to both MeHg and PUFA, we would expect the β for MeHg to be negative and the β for PUFAs to be positive. The relationship among IQ, MeHg exposure, and PUFA intake (in the absence of interaction) is described by a plane in three dimensions (Figure 3). The tilt of the regression plane in three dimensions is determined by the independent, unconfounded slopes (βs) of MeHg exposure and PUFA intake. In this example, as MeHg intake increases, PUFA intake would have to increase to maintain a mean IQ value in the population. Note also that in this hypothetical example, there is a level of MeHg intake at which no increase in PUFA could result in an increase of *x* IQ points above the mean score. Although there has been no consistent evidence of a MeHg–PUFA statistical interaction, such interactions may ultimately be present for one or more end points. If statistical interaction occurs, the relationship shown in Figure 3 becomes more complicated, but interaction can still be addressed in the approach we outline here. For the purposes of illustrating the principles of the approach, however, we assume the simple case of no statistical interaction.

**Figure 4:**
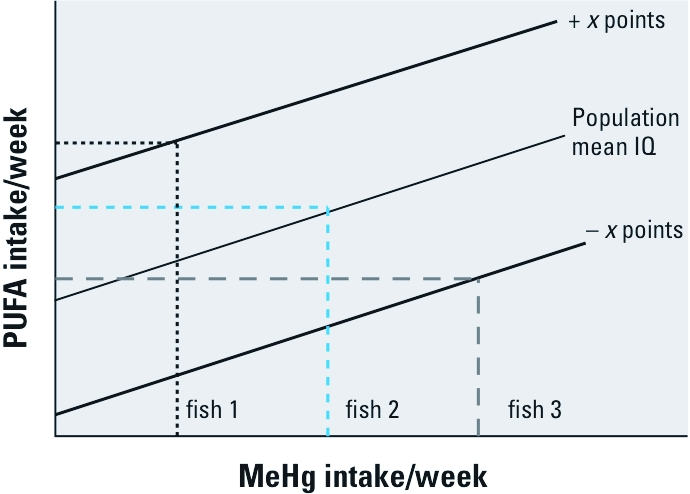
An example of how the same weekly intake of different species of fish with different characteristic MeHg and PUFA concentrations would influence IQ scores given a hypothetical regression relationship among IQ, MeHg exposure, and PUFA intake (see the description of this figure in the text for a more detailed explanation). *x* designates a specific, but indeterminate value.

This type of relationship allows us to predict the value for a dependent outcome variable, such as IQ, that would result from independent values of MeHg and PUFA. A given combination of MeHg exposure and PUFA intake can be chosen that reflects the characteristics of a particular consumption pattern for a specific species of fish, for example, 8 oz of largemouth bass per week. Data on MeHg [U.S. Food and Drug Administration (FDA) 2009] and PUFA concentrations (Mahaffey 2004) in commonly consumed species of fish are available. The MeHg and PUFA intake rates combined with their respective unconfounded β values would uniquely express the balance of risk and benefit that would result from that consumption pattern and species The same approach could also be applied to a diet that combines various species of fish by summing the MeHg and PUFA intakes. Figure 4 assumes that unconfounded βs specific to the relationship of MeHg and PUFA intake to IQ performance have been generated from the appropriate studies and that the combination of these fixed βs and variable levels of MeHg and PUFA intake deterministically influence IQ performance. Each of the solid lines represents a constant level of IQ performance (the population mean IQ and the mean ± *x* IQ points). Thus, each possible combination of MeHg and PUFA intake corresponds to a specific level of IQ performance. The dashed lines present three of these possible combinations, each corresponding to the MeHg and PUFA intake resulting from consumption of, for example, 8 oz/week of a hypothetical fish species (or a specific fish diet). For fish 1, with a relatively low MeHg concentration and a relatively high PUFA concentration, weekly intake of 8 oz/week would be expected to result, on average, in an increase in IQ score relative to the mean population score. On the other hand, for fish 3, with a relatively high MeHg concentration and a relatively low PUFA concentration, the same intake would, on average, result in a decrease in IQ score of equal magnitude. An appropriate consumption advisory for fish 3 might caution against consumption of 8 oz/week. In theory, such data can be combined in any combination to reflect the mix of MeHg and PUFA characteristics of any given species of fish or fish diet. The intake rate of that fish could then be adjusted to result in an overall benefit or, at least, the prevention of an overall negative outcome.

## Critical Caveats

Even if we consider only neurodevelopmental outcomes, many apparently discrete end points have been identified that are sensitive to MeHg. If these end points are indeed discrete, then there does not appear to be an *a priori* reason to assume that a MeHg–PUFA regression relationship derived for one of these end points will hold for any other. Thus, a range of end points should be evaluated, and advisories should be based on the end point most sensitive to MeHg risk. In addition, there is evidence that some end points respond negatively to increasing MeHg exposure but do not respond positively to increasing PUFA exposure (Budtz-Jørgensen et al. 2007; Choi et al. 2008; Lederman et al. 2008). If such suggestions are confirmed, end points adversely associated with MeHg without being positively associated with PUFA would seem to be the most sensitive end points, and risk–benefit advisories for such end points would collapse to the original approach of risk-only advisories. The overall picture of the relevant end points and their relative sensitivities to MeHg risk and PUFA benefit will likely emerge from a review of multiple studies.

An additional caveat arises from Strain et al. (2008). When both MeHg and the omega-3 PUFAs are controlled in the model of the 9-month and 30-month physical development index, the omega-6 PUFAs appear to have an adverse effect (negative β) similar to MeHg but opposite to the effect of the omega-3 PUFAs. Thus, even if we consider only risk–benefit balancing for MeHg and the PUFAs, it may be insufficient to merely balance the risks and benefits of MeHg and the omega-3 PUFAs. Future studies designed to generate the appropriate unconfounded data for MeHg and PUFAs should therefore include the omega-6 PUFAs in their design,

## Other Risk–Benefit Advisory Approaches for MeHg/PUFAs

Ginsberg and Toal (2009) derived a quantitative risk–benefit approach using empirical slopes of risk and benefit versus intake for myocardial infarction derived from Guallar et al. (2002) and a single neurodevelopmental end point, visual recognition memory score, derived from Oken et al. (2005). However, Oken et al. (2005) reported the rate of fish meals rather than PUFA per se, and the ability to appropriately control PUFA benefit for MeHg effects on that basis is not clear. In addition, as discussed above, reliance on the risk–benefit balance from a single neurodevelopmental end point may lead to consumption advice that is not protective for other end points. Nonetheless, the underlying approach presented by Ginsberg and Toal is consistent with the approach presented here.

Gochfeld and Burger (2005) conducted a careful search for cardiovascular and developmental thresholds and asymptotes for fish consumption per se and compared these with regulatory guidelines for MeHg adverse effects [the U.S. EPA RfD (2001) and the Agency for Toxic Substances Disease Registry (ATSDR) minimal risk level (MRL) (ATSDR 1999)]. The apparent thresholds and benefit asymptotes were compared in order to derive curves that express overall risk and benefit as a function of grams per day of fish consumption for fish with assumed MeHg concentrations. As discussed above, these benefits data are likely to be confounded by the adverse effects of co-occurring MeHg. Furthermore, the regulatory guidelines for MeHg derived from studies that did not control for PUFA effects are likely confounded by PUFA benefits.

## Steps Forward

To provide unconfounded independent risk and benefit data, future cohort studies of fish-consuming populations should include dietary-based estimates of MeHg and PUFA intake and/or biomarkers of PUFAs, omega-3 and omega-6, and MeHg exposure. This may require additional work on PUFA pharmacokinetic modeling. Intake estimates should be included in regression (or structural equation) models to produce adjusted estimates of benefit and risk. If PUFA–MeHg interactions occur, nonlinear relationships among PUFA and MeHg intakes and outcomes will need to be addressed. This approach should be applied to studies of neurological development and to studies of cardiovascular effects. Particularly for neurodevelopmental outcomes, it will be necessary to generate unconfounded risk and benefit relationships for multiple developmentally significant outcomes to identify those that are most sensitive to MeHg risk and least subject to PUFA benefit. Particular attention should be given to identifying end points that may experience risk but no compensating benefit.

## Conclusions

Evidence for the beneficial effects of PUFAs in fish for cardiovascular and developmental end points and for the subtle, subclinical adverse effects of MeHg has been accumulating for more than a decade. Initially, the tendency was to assume that either risk or benefit was the most important parameter when considering fish consumption advisories. There is now a growing recognition that the most useful fish consumption advisories need to simultaneously address both PUFA benefit and MeHg risk and that both are tightly linked through fish consumption. For at least some important end points, the risks and benefits are mutually confounding, and neither can be adequately understood in isolation. It appears that the only way to untangle this confounding is through statistical approaches that reveal the naked (unconfounded) risk and benefit coefficients (βs) for each end point. Subsequent recombination of the risk and benefit coefficients can then describe the overall risk–benefit profile of each species of fish for a given end point. However, close reading of the literature suggests that different end points have different coefficients for risk and benefit and that some end points may be adversely affected by MeHg without experiencing beneficial effects of PUFA. This implies that there may be no such thing as an *a priori* representative end point that can be used arbitrarily to construct risk–benefit guidance, even given unconfounded coefficients for PUFA and MeHg. At least for neurodevelopment, it may be necessary to generate PUFA and MeHg coefficients for each end point deemed to have independent public health significance to identify the most risk-sensitive end point for use in constructing fish consumption advisories.

We encourage future researchers to generate data that relate both MeHg and PUFA intake to a range of end points in a way that allows the risks and benefits to be statistically disentangled and recombined to facilitate generalizable consumption advice.
